# Synthesis, Properties and Adsorption Kinetic Study of New Cross-Linked Composite Materials Based on Polyethylene Glycol Polyrotaxane and Polyisoprene/Semi-Rotaxane

**DOI:** 10.3390/ma16165594

**Published:** 2023-08-12

**Authors:** Ana-Maria Resmerita, Alexandra Bargan, Corneliu Cojocaru, Aurica Farcas

**Affiliations:** “Petru Poni” Institute of Macromolecular Chemistry, 41A Grigore Ghica Voda Alley, 700487 Iasi, Romania; anistor@icmpp.ro (A.B.); cojocaru.corneliu@icmpp.ro (C.C.)

**Keywords:** supramolecular networks, polyrotaxane, cross-linking, morphology, adsorption kinetic

## Abstract

New composite materials were prepared via cross-linking of polyethylene glycol/2-hydroxypropyl-β-cyclodextrins polyrotaxane (PEG/HPβCD) and polyisoprene/HPβCD semi-polyrotaxane (PI/HPβCD SR) with 1,6-hexamethylene diizocyanate (HMDI). Advanced instrumental methods (such WAXS (wide angle X-ray scattering), AFM (atomic force microscopy), SEM (scanning electron microscopy), and thermal and dynamic vapor sorption) were employed for the structural, morphological and thermal characterization of the resulting composite materials. The roughness parameters calculated using AFM indicate a smoother surface for the composite material with 10 wt% of PI/HPβCD SR, denoting that a homogeneous film was obtained. SEM analysis reveals porous morphologies for both composite materials and the pore sizes increase with the increasing concentration of PI/HPβCD SR in the matrix. Dynamic vapor sorption/desorption measurements and type IV isotherms confirmed the hydrophilic and porous materials, which are in agreement with SEM analysis. The composite with a higher PI/HPβCD SR concentration in the matrix showed increased thermal stability than that of the pure cross-linked material. This material was further tested as a sorbent for methylene blue (MB) dye removal from an aqueous solution. The adsorption capacity of the composite film was found to be 2.58 mg g^−1^ at 25 °C.

## 1. Introduction

The development of new supramolecular networks based on cross-linked polyrotaxane structures has gained increased attention due to their slide-ring motions properties [1,2,3,4,5,6,7]. Therefore, when these materials are exposed to an external stress stimulus, the cross-linked junctions can slide along the polymer chain like a “pulley”, which is reflected in the enhancement of the mechanical properties [8,9,10]. Additionally, this intrinsic mobility of the cross-linked junctions allows for achieving uniform stress dispensation and actuation, which is why they are recommended for a variety of applications [11,12,13,14]. To date, the most commonly designed slide-ring supramolecular networks employed the use of poly(ethylene glycol) (PEG) and α-cyclodextrins (α-CD) [15]. Recently, our group succeeded to obtain rotaxane structures using HPβCD, which also have the advantages of low cost, non-toxicity, and commercial availability [16,17]. For the construction of such supramolecular structures, the threading method is employed but in case of polymers with molecular weights higher than 30 kDa, coverage ratio is difficult to control. Despite this drawback, recently, it was proven that even at a lower coverage ratio the mobility of the encapsulated polymer in the supramolecular networks, as well as the mechanical properties, is maintained. These types of materials have found applicability as gas separation membrane [18]. This significant finding provides new opportunities for the synthesis of new composite materials. These new results enable us to extend the field of application from optoelectronics to dye adsorption from wastewater. The reason for this interest was that the adsorption of dyes on solid surfaces is an increasing demand for industrial applications. Despite the beneficial use of the dyes in the industry, their presence in wastewater was proven to be harmful to humans and the environment [19]; thus, their removal is required. Materials such as activated carbon [20,21], magnesium ferrite [22], or polymer macro-nanoparticles [23,24] have been tested as adsorbents. Efforts are being made to develop new alternative adsorption techniques, which are also inexpensive, for applying in wastewater treatment. Therefore, we propose here new composite materials that combine the versatility of the PEG polymers with the PI’s biorenewable properties as well as its biocompatibility [25,26].

Herein, we report the synthesis of two composite materials with PEG/HPβCD polyrotaxane and 5 and 10 wt% of PI/HPβCD SR obtained via cross-linking reaction using HMDI. The PEG/HPβCD polyrotaxane was end-capped by bulky triphenylmethane groups at both PEG ends, whereas N-benzylidene benzylamine end-capped only one end of the PI/HPβCD SR backbones.

These materials were completely characterized from physico-chemical and morphological points of view and the results were compared to those of the pure cross-linked material. Moreover, the composite with 10 wt% of PI/HPβCD SR was preliminarily tested as a sorbent for the adsorption of methylene blue dye (MB) at the solid/liquid interface.

## 2. Materials and Methods

### 2.1. Materials

2-hydroxypropyl-β-cyclodextrins (HPβCD) DS = 4.5 (Cyclolab, Budapest, Hungary 98%), bromotriphenylmethane (98% purchased from Alfa Aesar, Haverhill, MA, USA), ultrapure water (Millipore, resistivity = 18 MΩ cm), anhydrous tetrahydrofuran (THF) and dimethyl formamide (DMF) from Sigma-Aldrich (St. Louis, MA, USA), and 1,6-hexamethylene diizocyanate (HMDI) (99%, Aldrich, St. Louis, MA, USA) were purchased.

### 2.2. Characterization

^1^H NMR spectra were recorded at 25 °C on a Bruker Avance NEO 400 MHz (Bruker, Rheinstetten, Germany) instrument equipped with a 5 mm QNP (Quattro Nucleus Probe) direct detection probe and z-gradients. Samples were prepared in D_2_O or THF-d_8_ solvents. The solvent signal was used as the internal reference and the chemical shifts were reported as δ values (ppm). Wide angle X-ray scattering (WAXS) was performed using Shimadzu LabX XRD-6000 diffractometer (Columbia, MD, USA) with a Cu Kα radiation (λ = 1.54059 Å) and recorded in a 2θ range 2–80°. The scanning angle step was 0.02° and the scanning speed was 0.5 deg min^−1^. The attenuated total reflectance-Fourier transform infrared (ATR-IR) spectra were carry out on a Bruker Vertex 70 (Bruker, Rosenheim, Germany) at a resolution of 2 cm^−1^ in the range of 4000 to 600 cm^−1^, with an average of 64 scans. The swelling degrees of the materials were analyzed in terms of the mass retained at equilibrium. The samples were allowed to expand in DMF at room temperature for 7 days. Each film was wiped with filter paper to remove the solvent, and then the samples weights were estimated until a constant mass was obtained. The swelling ratio was calculated using the method that we previously reported [11]. Atomic force microscopy was employed to analyze the microstructure and the roughness parameters of the composite materials. The experiments were performed on an NTEGRA multifunctional Scanning Probe Microscope (NT-MDT Spectrum Instruments, Zelenograd, Moscow, Russia), using the software Nova 1.1.1.19891 (NT-MDT Spectrum Instruments Zelenograd, Moscow, Russia) for acquisition and data processing. The morphologies of the films were registered on areas of 10 × 10 µm^2^, in tapping mode using a NSG10 cantilever (tip curvature radius-10 nm, resonant frequency-183 kHz) from TipsNano (Tallinn, Estonia). Scanning electron microscopy (SEM) micrographs were obtained with Verios G4 UC Scanning Electron Microscope (Thermo Fisher Scientific, Waltham, MA, USA) equipped with an energy dispersive spectrometer (EDS, EDAX Octane Elite, Thermo Fisher Scientific, Waltham, MA, USA). The measurements were performed on an accelerating voltage of 20 kV, with a Large Field Detector (LFD) in low Vacuum Mode. All composite films were analyzed before and after swelling in DMF. The films subjected to the swelling process were freeze-dried prior to being analyzed via SEM. The dynamic water vapors sorption/desorption capacity of the samples was measured in a dynamic regime, using an automated gravimetric analyzer IGAsorp developed by Hiden Analytical (Warrington, UK). The ultrasensitive microbalance of this device allows for measuring the samples weight changes with the variation in humidity in the chamber at a constant temperature. Samples were placed in the measuring chamber and then dried at 25 °C in N_2_ atmosphere (250 mL min^−1^) until the equilibrium weights was reached at a relative humidity (RH) lower than 1%. The RH was gradually increased from 0 to 90%, with 10% humidity steps followed by a stabilization time from 40 to 60 min to obtain the sorption equilibrium for each step. Thermogravimetric analysis (TGA) was analyzed on a Mettler Toledo TGA/SDTA 851e equipment (Mettler Toledo, Greifensee, Switzerland) under constant N_2_ flow (20 cm^3^ min^−1^) with a heating rate of 10 °C min^−1^ in the range 25 to 800 °C. Differential scanning calorimetry (DSC) analyses were performed with a Mettler Toledo DSC-1 calorimeter (Mettler Toledo, Greifensee, Switzerland) under N_2_ atmosphere, using the following protocol: heating from −60 to 200 °C, cooling from 200 to −60 °C at 10 °C min^−1^ rate and then repeating the heating–cooling cycles. Data were collected from the second heating–cooling cycles. STAR^e^ software (Version 9.10, Mettler Toledo, Giessen, Germany) has been used to process the TGA and DSC curves. The adsorption experiments of MB cationic dye were carried out at 25 °C. The concentration of the cationic dye in the aqueous solution was determined with a double-beam UV-visible spectrophotometer (SPECORD Plus, Jena, Germany) based on performed calibration curves and absorption bands identification at λ_max_ = 667 nm. Exactly 50 mL of MB of initial concentration C*_i_* = 5 mg L^−1^ with a pH = 6.5 were stirred at 200 rpm; then, 0.0123 g of sorbent dose (SD) was immersed into the solution and kept for a specific period of time (240 min). The time of the experiments was adequately chosen in order to prevent MB dye photodegradation in an aqueous solution under visible light. The kinetic studies on MB photodegradation confirmed that after 240 min of being exposed to visible light, the concentration of the dye decreases by 5% from the total concentration [27,28]. Aliquots were taken from the dye solution at different contact times and the concentration of MB was determined via UV-vis (ultraviolet visible spectroscopy) at λ_max_ = 667 nm. The adsorption capacity as a function of time was calculated according to Equation (1):

(1) $$q_{t}\;\left(\frac{mg}{g}\right)=\frac{\left(C_{0}-C_{t}\right)\;V}{m\cdot 1000}$$

where *q_t_* (mg g^−1^) represents the adsorption capacity, i.e., amount of dye per weight of sorbent, which is absorbed in time *t* and reaches the higher value *q_e_* when the adsorption process reaches equilibrium. *C*_0_ and *C_t_* (mg L^−1^) are the initial and the final concentrations of MB in the solution, respectively; *V* (mL) is the volume of the working solution and *m* (g) is the weight of the composite film SD. The sorbent was then subjected to a desorption experiment in which the spent sorbent of 0.01 g was immersed in 2 mL of DMF as eluent for one hour.

### 2.3. Synthesis

#### 2.3.1. Synthesis of Diamine Polyethylene Glycol 

PEG-diamine with M_n_ = 35 kDa, calculated via ^1^H NMR(Proton Nuclear Magnetic Resonance), was synthesized using the method previously reported [29].

#### 2.3.2. Synthesis of PEG/HPβCD

The PEG/HPβCD was obtained according to a previously reported procedure [16]. The coverage ratio of the polyrotaxane was calculated by integration of the area (*I_H_*^6′^/13.5)/(*I_a_*/3181), where *I_a_* is the integral of PEG protons (3.71 ppm) and *I_H_*^6′^ is the integral of HPβCD protons (1.17–1.15 ppm). The coverage ratio was found to be 4% and the *M_n_* of about 50 kDa was calculated using ^1^H NMR (Figure S1).

^1^H NMR (400 MHz, D_2_O, ppm): δ = 7.89–7.74 (H b, c and d), 5.70–5.09 (H^1^, HPβCD), 4.03–3.85 and 3.53 (m, H^2−6^, HPβCD), 3.71 (s, Ha of PEG-chain), and 1.17–1.15 (d, H^2′−6′^,-CH_3_ from hydroxypropyl groups).

#### 2.3.3. Synthesis of PI/HPβCD SR

The synthesis of PI/HPβCD semi-polyrotaxane was performed according to the previously reported procedure [17].

#### 2.3.4. Synthesis of the Pure Cross-Linked Material 1

The material (**1**) was obtained by mixing the solution of PEG/HPβCD (50 mg dissolved in 3 mL of dry DMF) with HMDI (0.2 mL, 1.24 mmol). The mixture was vigorously stirred for 1 h at ambient temperature, and then the temperature was increased to 80 °C and maintained for 27 h. Then, the reaction mixture was cast onto a Teflon Petri dish and placed into the oven at 120 °C overnight to evaporate the solvent. A solid state film was obtained with 0.16 mm thickness. The film was purified by dipping in DMF for five days in order to remove the unreacted components, and then dried in the oven at 100 °C for 24 h.

#### 2.3.5. Synthesis of the Cross-Linked Materials 2 and 3

In a solution mixture of dried THF/DMF 3/1 *v/v*, 5 or 10 wt% of PI/HPβCD SR were added, followed by the addition of 50 mg of PEG/HPβCD solubilized in 1 mL of DMF. HMDI, the cross-linker (0.1 mL, 1.25 mmol), was added and the reaction mixture was stirred for 1 h at ambient temperature; then, the temperature was increased at 40 °C and maintained for 48 h. The solutions were cast onto a Teflon Petri dish and dried in the oven at 120 °C for 24 h to obtain solid-state films. After purification steps by dipping in water and DMF, composites materials as solid films were obtained with 0.16 mm thickness.

## 3. Results and Discussion

**Scheme 1:**
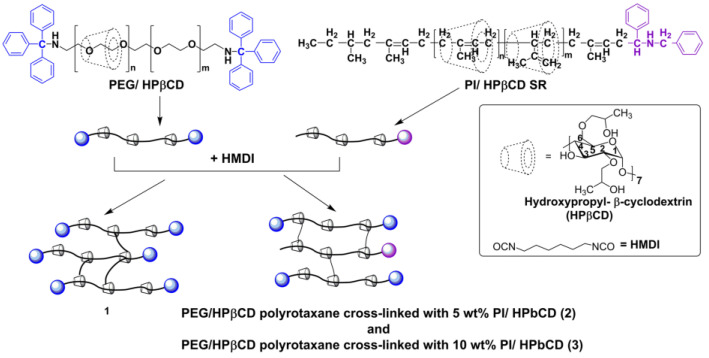
The synthesis of the composite materials.

This paper describes the synthesis and characterization of two new composite materials, 2 and 3, based on PEG/HPβCD polyrotaxane cross-linked with 5 and 10 wt% of PI/HPβCD SR, respectively. For comparison, the pure cross-linked film 1 was obtained via cross-linking of only the PEG/HPβCD polyrotaxanes chains. The schematic representation of the synthesis of these composite materials is shown in Scheme 1.

### 3.1. Chemical and Physical Properties of the Composite Materials

#### 3.1.1. Chemical Analysis

**Figure 1:**
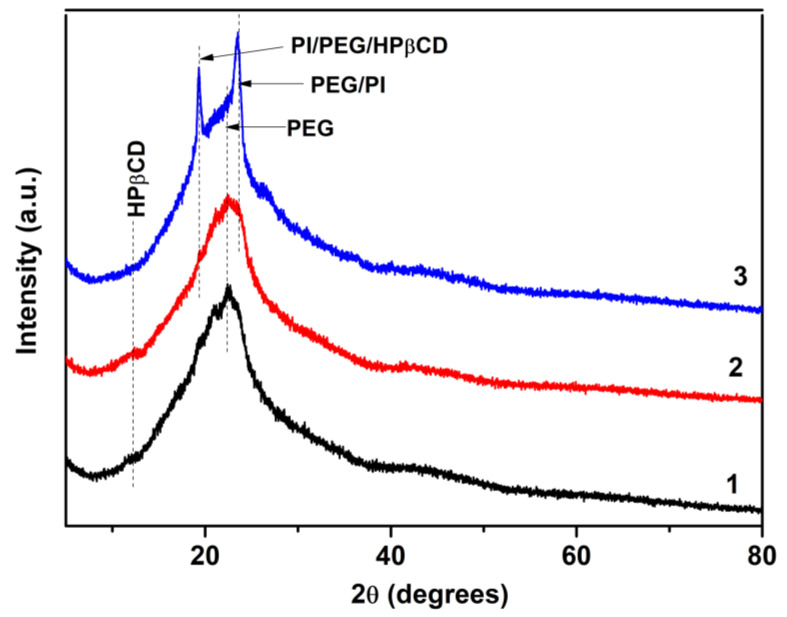
WAXS patterns of the **1**, **2** and **3** cross-linked materials.

For the chemical characterization of the composite materials, WAXS diffraction and ATR-IR spectroscopy were employed. The WAXS pattern of the pure cross-linked film **1** showed an amorphous halo with broad peaks in the low-angle regions 2θ = 12.22° and 22.7° (Figure 1). The peak in the 2θ = 12.22° region can be correlated with the disordered packing of HPβCD correlated with the steric hindrance of the hydroxypropyl groups [16,30]. The peak in the 2θ = 22.7° region is assigned to the short-range order of uncovered PEG segments of the cross-linked polymer matrix. The diffractogram of the composite material **2**, containing 5 wt% of PI/HPβCD SR in the matrix, exhibits the same amorphous halo with the peaks in the low-angle regions at 2θ = 12.22° and 22.7°, respectively; they also correlated to the disordered packing of HPβCD and short-range order of uncovered PEG segments [31]. The peaks corresponding to the PI polymer are overlapped with the ones from the PEG matrix [17,32,33]. In contrast, the diffraction pattern of the composite material **3**, containing 10 wt% of PI/HPβCD, exhibits more intense peaks at 2θ = 19.32° and 23.53°, respectively. These peaks could be attributed to the mutual influence between PEG/HPβCD and PI/HPβCD SR, resulting in a slight increase in the crystallinity, which is in accordance with the DSC results. From the results obtained above, we can conclude that even if PEG backbones only have a 4% coverage ratio with HPβCD, chain crystallization is suppressed.

Additional information regarding the chemical structures of these composite materials was obtained via ATR-IR spectroscopy. The IR spectrum of the pure cross-linked film **1** (Figure S2) shows absorption bands located at 3329 cm^−1^, which is attributed to the N-H stretching of the HMDI cross-linker overlapped with the O-H bands of HPβCD at 2925 cm^−1^ (C-H stretching vibration of HPβCD). The bands at 2860 cm^−1^ are assigned to the C-H stretching vibration of PEG, whereas the bands at 1621 and 1538 cm^−1^ are associated with the N-H out of plane bending and C-N stretching from HMDI. The IR spectrum showed additional bands located at 1244 cm^−1^ (C-N and C-O stretching vibrations urethane group) and 1095–1031 cm^−1^ (C-C or C-O stretching of HPβCD). The IR spectra of compounds **2** and **3** showed all the characteristic bands of PEG/HPβCD polyrotaxane overlapped with the ones corresponding to PI/HPβCD SR.

#### 3.1.2. Physical and Morphological Properties of the Materials

**Figure 2:**
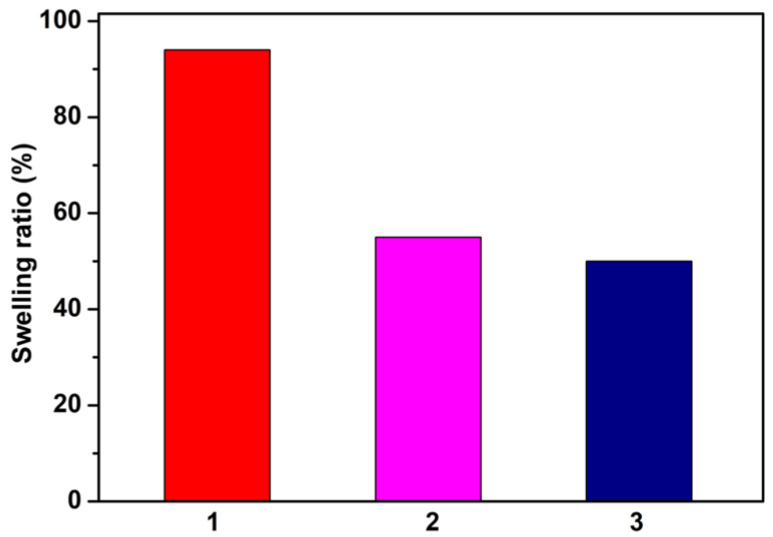
Swelling ratio of the composite materials in DMF.

Further insights into the physical properties of these composite materials were obtained by analyzing the swelling behavior in DMF (Figure 2). It was found that a higher solvent uptake was obtained for the pure cross-linked film **1**, while for the composite materials **2** and **3**, the swelling ratio decreases with increasing PI/HPβCD SR concentration in the matrix. We supposed that the flexibility of the cross-linked network is affected by the conformational changes in the PI chains in the PEG/HPβCD matrix [34].

**Figure 3:**
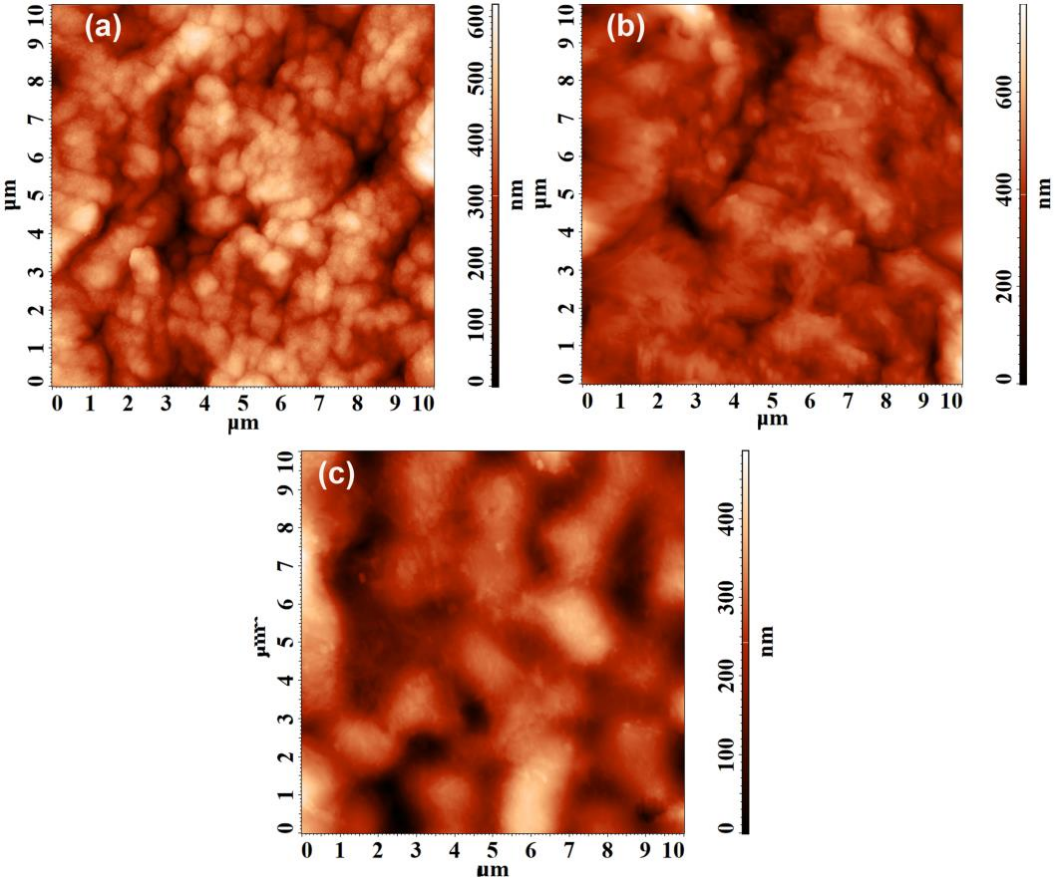
Two-dimensional AFM surface topography of the 10 × 10 µm^2^ area of **1** (**a**), **2** (**b**) and **3** (**c**) composite materials.

The surface morphologies of the composite films were also investigated via AFM and SEM microscopies. AFM microscopy was used to analyze the phase and surface topography of composites films in order to prove the presence of PI/HPβCD SR in the PEG matrix. The 2D topography images and roughness parameters are shown in Figure 3 and summarized in Table 1. The surface pattern of the pure cross-linked material **1** indicated microphase separation, which may be attributed to the packing of the PEG uncovered segments and the PEG segments encapsulated into HPβCD. The surface of the pure cross-linked material appeared to be more rough, with average roughness (S*_a_*) and root-mean-square roughness (S*_q_*) values of 70.69 and 89.02 nm, respectively. In contrast, the surfaces of the composite materials **2** and **3** appeared to be smoother after the incorporation of PI/HPβCD SR into the matrix. Additionally, with the increased concentration of PI/HPβCD SR, the S*_q_* values decreases from 64.73 nm in the composite material **2** to 58.16 nm in case of the composite material **3**. This result is an indication that the presence of PI/HPβCD SR has a beneficial effect in obtaining more homogeneous composite materials.

**Figure 4:**
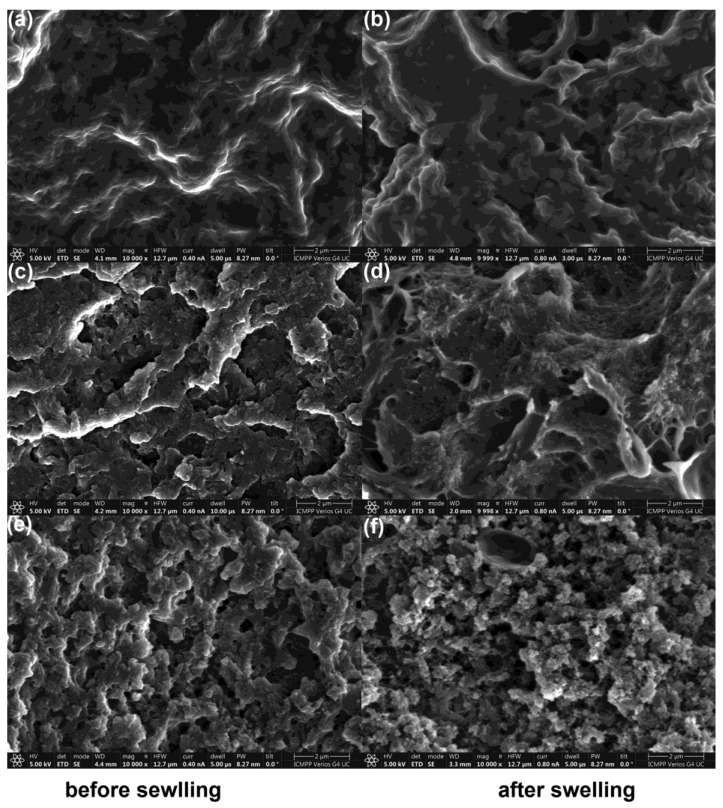
SEM micrographs showing cross-sectional views of the cross-linked materials **1** (**a**,**b**), **2** (**c**,**d**) and **3** (**e**,**f**) before and after swelling in DMF.

SEM images were also taken to analyze the morphology of the materials, both on the surface and in the cross-sectional view. Taking into account the swelling behavior of the films, SEM analysis was performed before and after swelling in DMF (Figure S3 and Figure 4). The surface view of the composite films exhibited a compact, dense, and nonporous structure, indicating good homogeneity and compatibility between both complex structures (Figure S3), while for the pure cross-linked film **1,** a phase separation was observed, this result being in accordance with the AFM analysis. The cross-sectional view of the pure cross-linked film showed a dense and nonporous morphology (Figure 4). In case of the both composite materials with PI/HPβCD SR, the morphology changes and became more porous. In addition, the density of the pores increases with increasing concentration of PI/HPβCD SR in the matrix. The average pore sizes of the composite materials were estimated using the image J analysis software and the values were obtained after a statistical correction was applied [11]. The average pore diameters of the composite materials **2** and **3** before swelling were found to be 0.020 and 0.038 μm, respectively. After the swelling process, the pores values of films **2** and **3** were found to be 0.036 and 0.044 μm, respectively. This result indicated that with the increasing concentration of PI/HPβCD in the matrix, the porosity increases following the same trend identified in the case of the polystyrene/semi-rotaxane composite films [11]. These findings could be attributed to either the conformational changes in the polyrotaxanes as a result of their physical interactions or to the increased crystallinity generated via the PI chains, which was also found in the WAXS analysis. 

**Figure 5:**
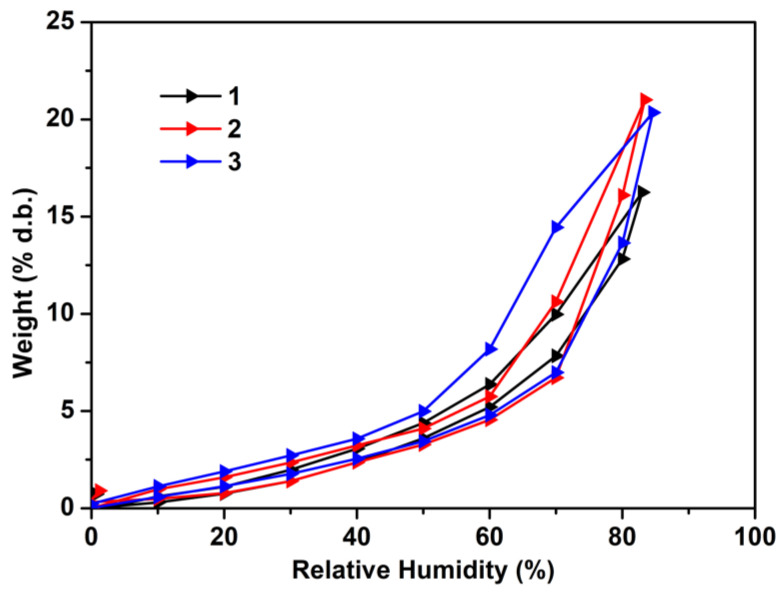
Water vapor sorption/desorption isotherms of the pure cross-linked film (**1**) and the films of composite materials **2** and **3**.

Taking into account that the PI/HPβCD SR film surface is hydrophilic [17] and it induces porous morphologies, it is expected that the composite films should have hydrophilic surfaces. To obtain more insights into the surface morphological characteristics, dynamic vapor sorption measurements were further performed. The results are presented in Figure 5 and summarized in Table 2.

According to the IUPAC (International Union of Pure and Applied Chemistry) classification [35], the shape of the water sorption curves was correlated to type IV isotherms. As observed in Figure 5, the isotherms have hysteresis, which are characteristic of the mesoporous and hydrophilic surfaces. The shape sorption/desorption isotherms indicated a reduced water vapor sorption at lower values of relative humidity (RH) and a sharp increase in water sorption at RH values closer to 100%. As can be seen, the composite materials have similar sorption capacity values to that of the pure cross-linked film **1** and were attributed to the compact surface morphologies of the materials. Analysis showed that the surface area efficiency available for adsorption in the case of material **2**, containing 5 wt% of PI/HPβCD SR, is lower compared to those of the materials **1** and **3**. To provide further evidence, the Brunauer–Emmett–Teller (BET) kinetic model [36] was applied and it was found that the composite materials have moderately high surface area, confirming their micro porosity. The pore sizes estimated using BET are not in agreement with the values obtained from SEM analysis due to the fact that the kinetic model estimates a cylindrical geometry of the pores [34]. Overall, the result obtained from sorption/desorption analysis in water revealed important information regarding their potential applications.

#### 3.1.3. Thermal Analysis

**Figure 6:**
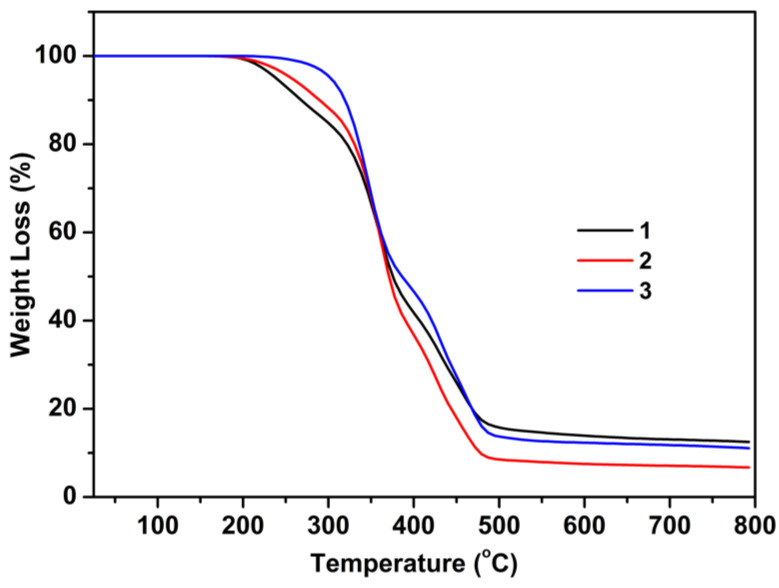
Thermograms of the cross-linked materials **1**, **2** and **3**.

The thermal properties of the composite materials were investigated via thermogravimetric analysis (TGA) and differential scanning calorimetry (DSC). As shown in Figure 6 and Figure S4 and Table 3, the TGA curve of the pure cross-linked film **1** exhibited three weight loss stages, starting from 205 °C. The composite material **2** with 5 wt% of PI/HPβCD SR in the matrix exhibited three weight loss stages as well, but the onset of decomposition appeared around 220 °C. The increased thermal stability of the composite material **2** proved the presence of PI/HPβCD SR in the matrix. Moreover, in the case of the composite material **3** with 10 wt% of PI/HPβCD SR in the matrix, the TGA curve shows only two weight loss stages, with the onset of decomposition around 309 °C. This result confirmed the presence of PI/HPβCD SR in the matrix, which proves to have a beneficial effect on thermal stability.

**Figure 7:**
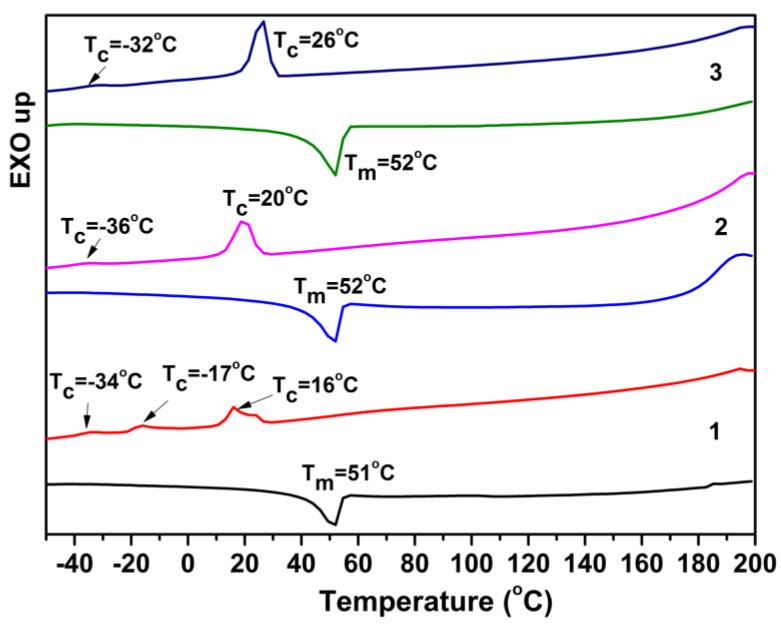
Second heating–cooling DSC curves of the cross-linked materials **1**, **2** and **3**.

The DSC results provided additional information to the TGA analysis. Hence, the second heating scan of the cross-linked materials (Figure 7) shows only an endothermic signal around 51 °C for **1**, whereas for **2** and **3**, the endothermic signal appear at 52 °C. Since these transition temperatures are near the melting point of the pure PEG, this process is consistent with the melting temperature of the uncovered segments via HPβCD of PEG backbones in the cross-linked matrix [30]. It is important to notice that in the temperature range of −60 °C to 200 °C no glass transition temperature of PI/HPβCD SR was identified, which is an indication of its homogeneity. Another observation is the presence of exothermic signals corresponding to the cold crystallization processes on the cooling scans. Thus, for the pure cross-linked film **1** three crystallization exothermic peaks were observed at −34 °C, −17 °C and 16 °C with the specific heat of (C_p_) 0.81 J g^−1^, 1.69 J g^−1^, and 5.57 J g^−1^, respectively. These crystallization temperatures are far from the melting temperatures and can be attributed to the short-range order of the uncovered PEG segments. Conversely, the composite materials exhibited only two exothermic signals attributed to crystallization processes at −36 °C (C_p_ = 0.41 J g^−1^) and 20 °C (C_p_ = 9.79 J g^−1^) for the composite material **2**; in the case of the composite material **3**, it was at −32 °C (C_p_ = 1.05 J g^−1^) and 26 °C (C_p_ = 18.52 J g^−1^), which can be attributed to the PEG crystalline domain and also to the presence of PI/HPβCD SR in the cross-linked matrix. It is well known that the PI polymer has good flexibility but in the cross-linked systems at room temperature, it gains plastic properties [33].

### 3.2. Adsorption Kinetics

**Figure 8:**
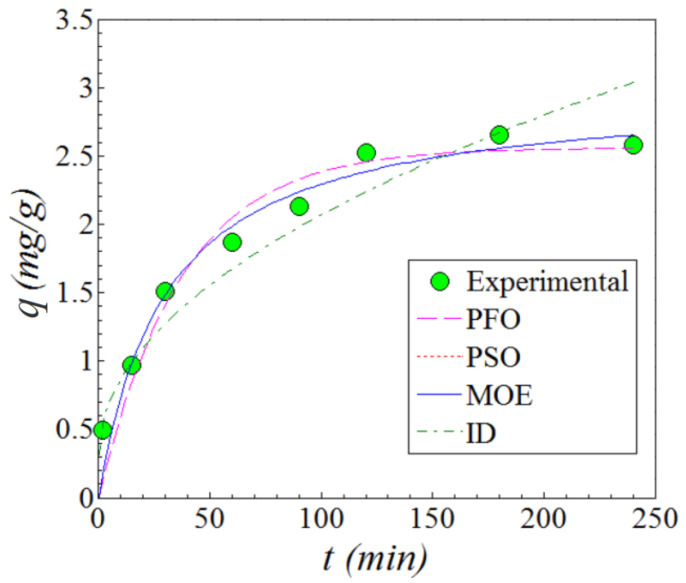
Adsorption kinetics of MB dye onto the composite material **3** under the following experimental conditions: T = 298 K, SD = 0.0123 g L^−1^, and [MB]_0_ = 5 mg L^−1^.

The adsorption kinetic was performed only on the composite material **3** with 10 wt% of PI/HPβCD SR incorporated into the PEG/HPβCD matrix, because this material displayed better surface morphologies properties and porosity. To identify the maximum adsorption of MB in water, the solutions with concentrations between 0.5 and 5 mg L^−1^ were firstly prepared and the calibration curve was represented (Figure S5). The adsorption of MB dye into the composite material against the contact time is represented in Figure 8, revealing a maximum of adsorption capacity equal to 2.58 mg g^−1^. As expected, the increase in the contact time led to an increase in the adsorption capacity of MB. The adsorption kinetic of MB exhibited a two-stage behavior, meaning that a faster increase was observed within the first 30 min (first stage), and then a slower and gradual adsorption increase (second stage) until the equilibrium is reached during the 240 min of the experiment. To investigate the adsorption mechanism, the kinetic experimental data were fitted to the pseudo-first-order kinetic model (PFO), the pseudo-second-order kinetics (PSO), the mix 1,2-order kinetics (MOE), and the intra-particle diffusion kinetics (ID) models [37,38,39,40]. In Table 4, the kinetic models and parameters adsorption are summarized: *q_t_* and *q_e_* represent the adsorption capacity at a given time and at equilibrium, respectively; *K*_1_ is the rate constant for the PFO model; *K*_2_ is the rate constant for the PSO equation; *K*_1_ and *K*_2_ are the rate constants for mixed 1,2-order kinetics (MOE); and *k_d_* is the rate constant for the intra-particle diffusion (ID) model. The goodness-of-fit between the calculated values *q^calc^* (from kinetic models) and the experimental values *q^obs^* was expressed via the residual error function *ε*^2^, representing the sum of the square of the errors (ERRSQ), which can be written as follows:

(2) $$\varepsilon^{2}=\sum_{j-1}^{n}{\left(q_{j}^{calc}-q_{j}^{obs}\right)}^{2}$$

Generally, the lower the values of the error function (*ε*^2^), the better the prediction of the model. By inspecting the *ε*^2^-values (Table 3), it can be inferred that the adsorption process obeyed the pseudo-second-order model (the PSO model was the best). This fact was also corroborated with the results of the MOE model, which revealed that *K*_2_ >> *K*_1_ (see Table 3). Thus, both PSO and MOE models provided the best accuracies in predicting the adsorption kinetics data. As shown in Figure 8, the estimations obtained via PSO and MOE are almost identical (predictions of MOE overlap with PSO).

Further, the desorption process was analyzed for the sorbent loaded with MB (Figure S6). Therefore, the sorbent was immersed for one hour in 2 mL DMF as eluent without stirring. As can be observed, the sorbent film returns to its initial color, which suggested that desorption of MB occurred. Additionally, we conducted three cycles of adsorption–desorption of MB in order to test the reusability of the material. The above experiments were performed using the same experimental conditions. The adsorption capacity after the third experiment decreased from 2.58 to 1.76 mg g^−1^. To further examine if changes in the morphology of the film occurred, AMF and WAXS were also performed. The 2D AFM image (Figure S7) showed a slight microphase separation, while the roughness parameter (Table S1) indicated that the surface became smoother in comparison to the initial material; the Sa decreases from 58.16 to 54.43 nm. The WAXS pattern (Figure S8) indicated a drop in diffraction intensities and a shift in the crystallinity peaks. The initial material exhibited reflection peaks at 2θ = 19.32° and 23.53°, while after the adsorption–desorption process, an intense peak is observed at 2θ = 11.98°, which may be attributed to an ordered packing of the polyrotaxanes in the supramolecular material, and an amorphous hallow at 2θ = 20.54°. From the obtained results, we can assume that this composite material based on the cross-linked rotaxanes architectures can be utilized for the adsorption of MB dye from aqueous solutions. This is a preliminary study and additional investigations are required in due course.

## 4. Conclusions

In summary, we report the synthesis of new composite materials based on PEG/HPβCD polyrotaxanes and PI/HPβCD SR structures. The composite materials contained different concentrations of PI/HPβCD SR and the effect of the later over matrix was investigated. WAXS and ATR-IR spectroscopies confirmed the chemical structure of the materials. AFM microscopy showed that with the increase in PI/HPβCD SR in the matrix, the roughness parameters are lower than the pure cross-linked film, (S*_a_* decreases from 70 nm to about 58 nm),which indicate the formation of more homogeneous surfaces. The SEM cross-sectional view showed porous structure and the pore diameter increase with the increase in PI/HPβCD SR in the matrix. The dynamic vapor sorption measurements proved the porous morphology observed via SEM. The material with 10 wt% of PI/HPβCD SR in the matrix exhibited an increase with 100 °C thermal stability. Moreover, this material was tested as a sorbent for MB dye adsorption from an aqueous solution. The obtained results demonstrated that the adsorption equilibrium data were well fitted to the pseudo-second-order kinetic model, disclosing a constant rate equal to 1.112 × 10^−2^ g mg^−1^ min^−1^. Desorption process from the spent sorbent of the dye was also tested in a lower volume of DMF solvent. For this reason, we propose the optimization of the composite material’s properties to increase the adsorption and desorption efficiencies, and for the possibility of multiple-cycle reuse of the spent sorbent. A future strategy is to develop the utilization of these cross-linked materials, and thus find additional improvements in the results presented here.

## Figures and Tables

**Table 1:** AFM surface roughness parameters of **1**, **2** and **3** composite materials.

Sample	S*_a_* (nm)	S*_q_* (nm)	S*_sk_*	S*_ku_*	S*_z_* (nm)
**1**	70.69	89.02	−0.382	0.157	308.94
**2**	64.73	86.08	−0.429	1.599	358.29
**3**	58.16	73.09	−0.125	0.040	239.54

S*_a_* = average roughness; S*_q_* = root-mean-square roughness; S*_sk_* = skewness of the topography height distribution; S*_ku_* = kurtosis of the topography height distributions; and S*_z_* = 10-point average of the absolute heights.

**Table 2:** Surface parameters based on adsorption/desorption isotherms.

Sample	W ^a^ [%]	r_pm_^b^ [nm]	BET ^c^	
			A ^d^ [m^2^ g^−1^]	W_m_^e^ [g g^−1^]
**1**	20.2272	3.97	101.944	0.0290
**2**	20.9997	4.70	89.250	0.0254
**3**	20.3485	4.04	100.842	0.0287

^a^ Water sorption capacity. ^b^ Average pore size.^c^ Determined based on the desorption branch of the isotherm (registered up to a relative humidity of 40%). ^d^ Specific surface area. ^e^ Weight of water forming monolayer.

**Table 3:** Thermogravimetric data of the composite materials.

Sample	Step	T_onset_^a^ [°C]	T_peak_^b^ [°C]	T_endset_^c^ [°C]	W ^d^ [%]	Rezidue ^e^ [%]
**1**	I	205	241	263	15.06	
	II	325	358	377	46.30	11.60
	III	419	445	477	27.04	
**2**	I	220	263	303	13.09	6.32
	II	329	360	376	49.53	
	III	410	426	471	31.06	
**3**	I	309	346	368	53.31	9.51
	II	413	455	483	37.18	

^a^ The starting temperature of the degradation process. ^b^ The maximum degradation temperature. ^c^ The temperature of complete degradation process. ^d^ The mass percentage loss recorded on each stage. ^e^ The residue at the end of degradation.

**Table 4:** Kinetic models and parameters for MB dye adsorption onto the composite material **3** under the following experimental conditions: T = 298 K, SD = 0.0123 g L^−1^, and [MB]_0_ = 5 mg L^−1^.

Model	Kinetic Model (Rate Equation)	Kinetic Model (Non-Linear Equation)	Kinetic Parameters
PFO ^a^	$\frac{dq_{t}}{dt}=k_{1}\left(q_{e}-q_{t}\right)$	$q_{t}=q_{e}\left(1-e^{-k_{1}t}\right)$	*q_e_* = 2.5588 (mg/g) *K*_1_ = 2.6781 × 10^−2^ *ε*^2^ = 0.2429
PSO ^b^	$\frac{dq_{t}}{dt}=k_{2}\;{\left(q_{e}-q_{t}\right)}^{2}$	$q_{t}=\frac{k_{2}q_{e}^{2}t}{1+k_{2}q_{e}t}$	*q_e_* = 2.9841 (mg/g) *K*_2_ = 1.112 × 10^−2^ *ε*^2^ = 0.1553
MOE ^c^	$\frac{dq_{t}}{dt}=\underset{i}{\sum }K_{i}{\left(q_{e}-q_{t}\right)}^{i}$	$q_{t}=q_{e}\frac{1-e^{\left(-k_{1}t\right)}}{1-\frac{K_{2}q_{e}}{K_{1}+K_{2}q_{e}}e^{\left(K_{1}t\right)}}$	*q_e_* = 2.9835 (mg/g) *K*_1_ = 1.4296 × 10^−5^ *K*_2_ = 1.112 × 10^−2^ *ε*^2^ = 0.1553
ID ^d^	$\frac{dq_{t}}{dt}=\frac{k_{d}}{2\sqrt{t}}$	$q_{t}=k_{d}\sqrt{t}+J$	*k_d_* = 0.1757 *J* = 0.3124 *ε*^2^ = 0.4125

^a^ PFO—pseudo-first-order kinetics; ^b^ PSO—pseudo-second-order kinetics; ^c^ MOE—mix 1,2-order kinetics; and ^d^ ID—intra-particle diffusion kinetics.

## Data Availability

All data are incorporated in the manuscript. No additional data are available for sharing.

## Supplementary Materials

The following supporting information can be downloaded at https://www.mdpi.com/article/10.3390/ma16165594/s1, Figure S1: 1H NMR spectrum of PEG/HPβCD polyrotaxane in D2O, Figure S2: ATR-IR spectra of the **1**, **2** and **3** cross-linked materials, Figure S3: SEM micrograph (surface view) of the **1** (a, b), **2** (c, d) and **3** (e, f) cross-linked materials before and after swelling in DMF, Figure S4: Derivative TG curves of the of the **1**, **2** and **3** cross-linked materials, Figure S5: UV-vis absorbance of MB solutions in water (left) and the graphical representation of calibration curve at 667 nm (right), Figure S6: The images of the **3** composite materials after 240 h (left) and after desorption process in DMF (right), Figure S7: 2D AFM surface topography over of 10 × 10 µm^2^ area of **3** composite material after three cycles of adsorption/desorption of MB; Table S1. AFM surface roughness parameters of **3** composite materials after three cycles of adsorption/desorption of MB; Figure S8: WAXS pattern of **3** composite material after three cycles of adsorption–desorption of MB.

## References

[1] Araki J., Ito K. (2007). Recent advances in the preparation of cyclodextrin-based polyrotaxanes and their applications to soft materials. Soft Matter.

[2] Inutsuka M., Inoue K., Hayashi Y., Inomata A., Sakai Y., Yokoyama H., Ito K. (2015). Highly dielectric and flexible polyrotaxane elastomer by introduction of cyano groups. Polymer.

[3] Kato K., Yasuda T., Ito K. (2013). Viscoelastic properties of slide-ring gels reflecting sliding dynamics of partial chains and entropy of ring components. Macromolecules.

[4] Dikshit K., Bruns C.J. (2021). Post-synthesis modification of slide-ring gels for thermal and mechanical reconfiguration. Soft Matter.

[5] Jiang Y., Zhang Z., Wang Y.-X., Li D., Coen C.-T., Hwaun E., Chen G., Wu H.-C., Zhong D., Niu S. (2022). Topological supramolecular network enabled high-conductivity, stretchable organic bioelectronics. Science.

[6] Ogawa M., Kawasaki A., Koyama Y., Takata T. (2011). Synthesis and properties of a polyrotaxane network prepared from a Pd-templated bis-macrocycle as a topological cross-linker. Polym. J..

[7] Zheng J., Yang Z., Sang Y., Zhang F., Zhang Y., Zhang P. (2022). A simple preparation method of rotaxane cross-linker based on γ-cyclodextrin and its application in hydrogel materials. Mater. Today Commun..

[8] Ito K. (2010). Slide-ring materials using topological supramolecular architecture. Curr. Opin. Solid State Mater. Sci..

[9] Wang S., Chen Y., Sun Y., Qin Y., Zhang H., Yu X., Liu Y. (2022). Stretchable slide-ring supramolecular hydrogel for flexible electronic devices. Commun. Mater..

[10] Wang W., Zhao D., Yang J., Nishi T., Ito K., Zhao X., Zhang L. (2016). Novel slide-ring material/natural rubber composites with high damping property. Scientific Report.

[11] Resmerita A.-M., Asandulesa M., Farcas A. (2022). Evaluation of the chemical, morphological and dielectric properties of supramolecular networks consisting of polyethylene glycol polyrotaxanes and polystyrene/semi-rotaxane with hydroxypropyl-β-cyclodextrins. Macromol. Chem. Phys..

[12] Xiong X., Chen Y., Wang Z., Liu H., Le M., Lin C., Wu G., Wang L., Shi X., Jia Y.-G. (2023). Polymerizable rotaxane hydrogels for three-dimensional printing fabrication of wearable sensors. Nat. Commun..

[13] Noda Y., Hayashi Y., Ito K. (2014). From topological gels to slide-ring materials. J. Appl. Polym. Sci..

[14] Harada A., Takashima Y., Nakahata M. (2014). Supramolecular polymeric materials via cyclodextrins-guest interactions. Acc. Chem. Res..

[15] Harada A., Li J., Nakamitsu T., Kamachi M. (1993). Preparation and characterization of polyrotaxanes containing many threaded a-cyclodextrins. J. Org. Chem..

[16] Resmerita A.-M., Asandulesa M., Bulai G., Farcas A. (2019). Novel supramolecular networks based on PEG and PEDOT cross-linked polyrotaxanes as electrical conductive materials. Eur. Polym. J..

[17] Resmerita A.-M., Silion M., Cojocaru C., Farcas A. (2022). Structural and morphological characterization of a new semi-polyrotaxane architecture based on 2-hydroxypropyl-β-cyclodextrins and polyisoprene. React. Funct. Polym..

[18] Kato K., Naito M., Ito K., Takahara A., Kojio K. (2021). Freestanding tough glassy membranes produced by simple solvent casting of polyrotaxane derivatives. ACS Appl. Polym. Mater..

[19] Munir M., Nazar M.F., Zafar M.N., Zubair M., Ashfaq M., Hosseini-Bandegharaei A., Ud-Din Khan S., Ahmad A. (2020). Effective adsorptive removal of methylene blue from water by didodecyldimethylammonium bromide-modified brown clay. ACS Omega.

[20] Kannan N., Sundaram M.M. (2001). Kinetics and mechanism of removal of methylene blue by adsorption on various carbons-a comparative study. Dyes Pigments.

[21] Güleç F., Williams O., Kostas E.T., Samson A., Stevens L.A., Lester E. (2022). A comprehensive comparative study on methylene blue removal from aqueous solution using biochars produced from rapeseed, whitewood, and seaweed via different thermal conversion technologies. Fuel.

[22] Ivanets A., Prozorovich V., Roshchina M., Sychova O., Srivastava V., Sillanpää M. (2022). Methylene blue adsorption on magnesium ferrite: Optimization study, kinetics and reusability. Mater. Today Commun..

[23] Maruthapandi M., Kumar V.B., Luong J.H.T., Gedanken A. (2018). Kinetics, isotherm, and thermodynamic studies of methylene blue adsorption on polyaniline and polypyrrole macro-nanoparticles synthesized by c-dot-initiated polymerization. ASC Omega.

[24] Sultana S., Mohammad R., Khan Z., Umar K. (2012). Synthesis and characterization of copper ferrite nanoparticles doped polyaniline. J. Alloys Compd..

[25] Sundararajan S., Samui A.B., Kulkarni P.S. (2017). Versatility of polyethylene glycol (PEG) in designing solid-solid phase change materials (PCMs) for thermal management and their application to innovative technologies. J. Mater. Chem. A.

[26] Ates B., Koytepe S., Ulu A., Gurses C., Thakur V.K. (2020). Chemistry, structures, and advanced applications of nanocomposites from biorenewable resources. Chem. Rev..

[27] Pascariu P., Cojocaru C., Homocianu M., Samoila P., Grecu I., Bele A. (2022). New composite membranes based on PVDF fibers loaded with TiO_2_: Sm nanostructures and reinforced with graphene/graphene oxide for photocatalytic applications. Surf. Interfaces.

[28] Bushra R., Shahadat M., Ahmad A., Nabi S.A., Umar K., Oves M., Raeissi A.S., Muneer M. (2014). Synthesis, characterization, antimicrobial activity and applications of polyanilineTi(IV)arsenophosphate adsorbent for the analysis of organic and inorganic pollutants. J. Hazard. Mater..

[29] Resmerita A.-M., Assaf K.I., Lazar A.I., Nau W.M., Farcas A. (2017). Polyrotaxanes based on PEG-amine with cucurbit[7]uril, α-cyclodextrin and its tris-*O*-methylated derivative. Eur. Polym. J..

[30] Inomata A., Sakai Y., Zhao C., Ruslim C., Shinohara Y., Yokoyama H., Amemiya Y., Ito K. (2010). Crystallinity and cooperative motions of cyclic molecules in partially threaded solid-state polyrotaxanes. Macromolecules.

[31] Mayumi K., Ito K., Kato K., Gale P., Steed J. (2016). Polyrotaxanes and Slide-Ring Materials (Monographs in Supramolecular Chemistry No. 15).

[32] Wang Q., Ma Y., Meng J., Liu X., Xia L. (2022). Thermal-triggered Trans-1, 4 polyisoprene/polyethylene wax shape memory and self-healing composites. Polym. Test..

[33] Zhao H., Zhang C., Yang B., Zhang X., Dong X., Wang D., Liu G. (2023). Achieving high elasticity of trans-1,4-polyisoprene with a combination of radiation crosslinking and thiol-ene grafting. Polym. Chem..

[34] Watanabe H., Chen Q., Kawasaki Y., Matsumiya Y., Inoue T., Urakawa O. (2011). Entanglement dynamics in miscible polyisoprene/poly(p-tert-butylstyrene) blends. Macromolecules.

[35] Thommes M., Kaneko K., Neimark A.V., Olivier J.P., Rodriguez-Reinoso F., Rouquerol J., Sing K.S.W. (2015). Physisorption of gases, with special reference to the evaluation of surface area and pore size distribution (IUPAC Technical Report). Pure Appl. Chem..

[36] Brunauer S., Emmett P.H., Teller E. (1983). Adsorption of gases in multimolecular layers. J. Am. Chem. Soc..

[37] Othman N.H., Alias N.H., Shahruddin M.Z., Bakar N.F.A., Him N.R.N., Lau W.J. (2018). Adsorption kinetics of methylene blue dyes onto magnetic graphene oxide. J. Environ. Chem. Eng..

[38] Harrou A., Gharibi E., Nasri H., Ouahabi E.M. (2020). Thermodynamics and kinetics of the removal of methylene blue from aqueous solution by raw kaolin. SN Appl. Sci..

[39] Gürses A., Doğar Ç., Yalçına M., Açıkyıldız M., Bayrak R., Karaca S. (2006). The adsorption kinetics of the cationic dye, methylene blue, onto clay. J. Hazard. Mater..

[40] Enache A.-C., Samoila P., Cojocaru C., Apolzan R., Predeanu G., Harabagiu V. (2023). An eco-friendly modification of a walnut shell biosorbent for increased efficiency in wastewater treatment. Sustainability.

