# Supplementary material for: Synthesis, Properties and Adsorption Kinetic Study of New Cross-Linked Composite Materials Based on Polyethylene Glycol Polyrotaxane and Polyisoprene/Semi-Rotaxane
Source: Materials (Basel). 2023 Aug 12;16(16):5594. doi: 10.3390/ma16165594 (PMC10456596; doi:10.3390/ma16165594)
Supplement: Supplementary file 1 [file materials-16-05594-s001.zip › materials-2540758-supplementary.pdf]

materials

Synthesis, Properties and Adsorption Kinetic Study of New Cross-Linked Composite Materials Based on Polyethylene Glycol Polyrotaxane and Polyisoprene/Semi-Rotaxane

Ana-Maria Resmerita *, Alexandra Bargan, Corneliu Cojocaru and Aurica Farcas *

"Petru Poni" Institute of Macromolecular Chemistry, 41A Grigore Ghica Voda Alley, 700487 Iasi, Romania; anistor@icmpp.ro (A.B.); cojocaru.corneliu@icmpp.ro (C.C.)

* Correspondence: resmerita.anamaria@icmpp.ro (A.-M.R.); afarcas@icmpp.ro (A.F.); Tel.: +40-232-217-454.

Supplementary material

Table of Contents

1. Characterization of the materials......2

1.1. ^1H NMR spectrum of PEG/ HPβCD polyrotaxane in D_2O 2

1.2. FTIR characterization of the 1 material and 2 and 3 composite materials 2

1.3. SEM images surface view of the composite materials......3

1.4. The thermogravimetric analysis......3

1.5. UV-vis absorbance of MB and the calibration curves......3

1.6. Desorption process of MB from the sorbent in DMF......3

1.7. The 2D AFM topography of the 3 composite after the third cycle of adsorption-desorption......4

1.8. WAXS pattern of the 3 composite after the third cycle of adsorption-desorption......5

1. Characterization of the materials

1.1. ^{1}H NMR spectrum of PEG/ HPβCD polyrotaxane in D_2O

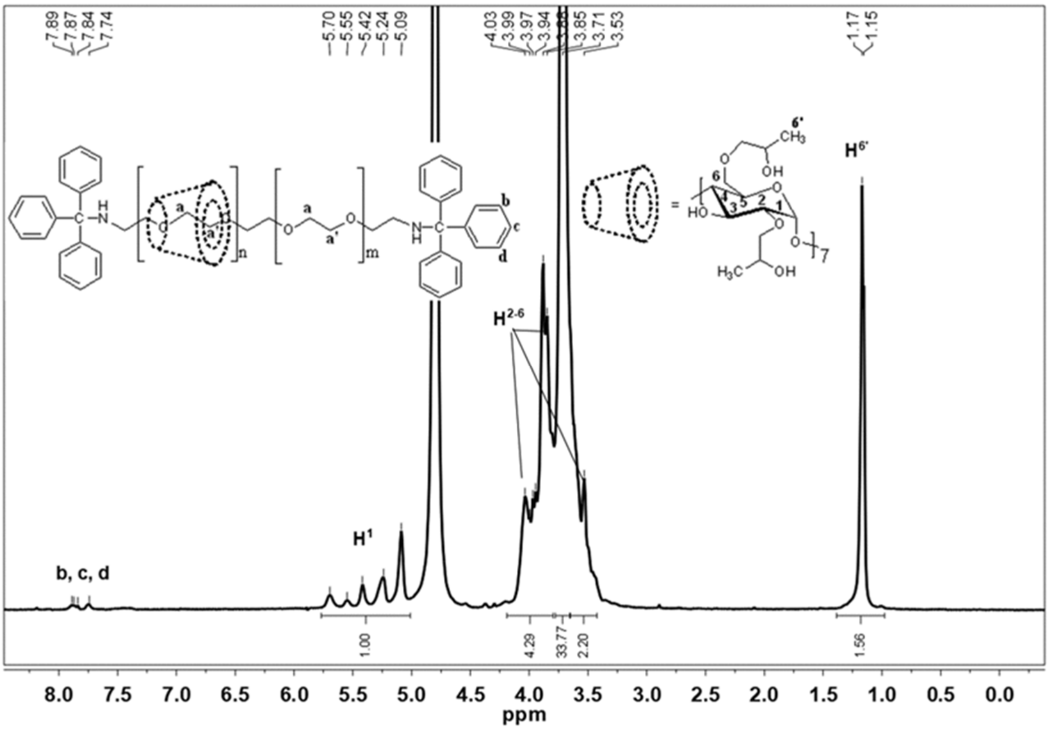

Figure S1. ^{1}H NMR spectrum of PEG/ HPβCD polyrotaxane in D_2O.

1.2. FTIR characterization of the 1 material and 2 and 3 composite materials

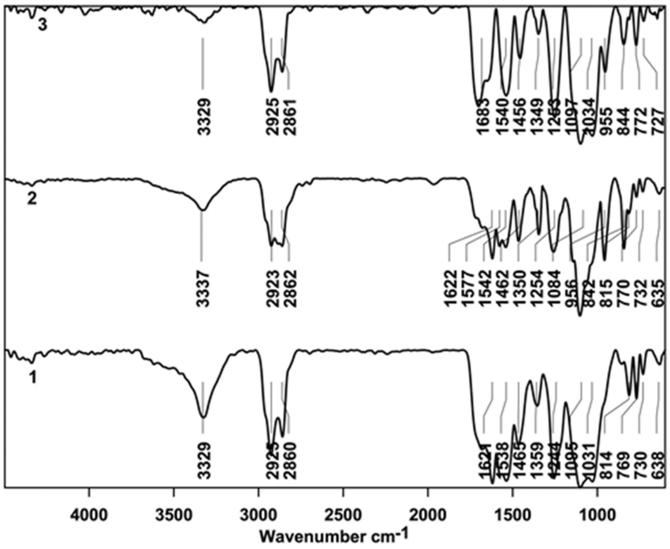

Figure S2. ATR-IR spectra of the **1**, **2** and **3** cross-linked materials.

1.3. SEM images surface view of the composite materials

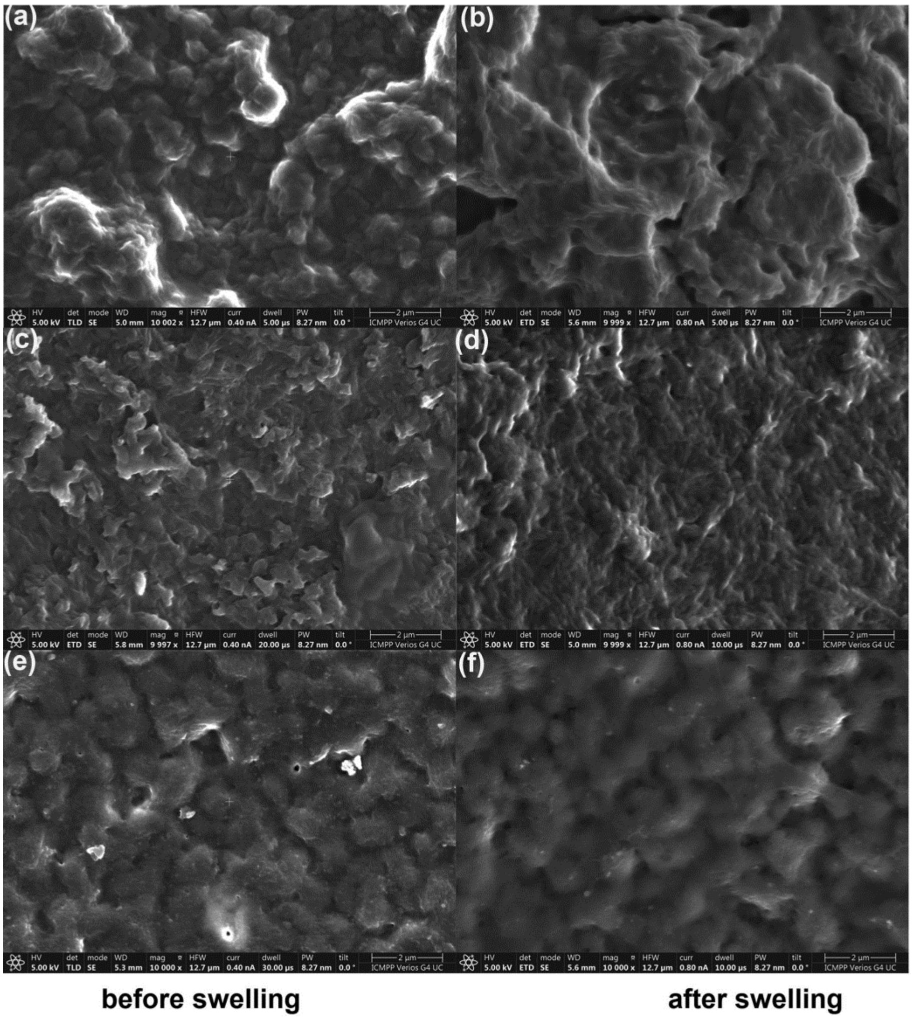

Figure S3. SEM micrograph (surface view) of the **1** (a, b), **2** (c, d) and **3** (e, f) cross-linked materials before and after swelling in DMF.

1.4. The thermogravimetric analysis

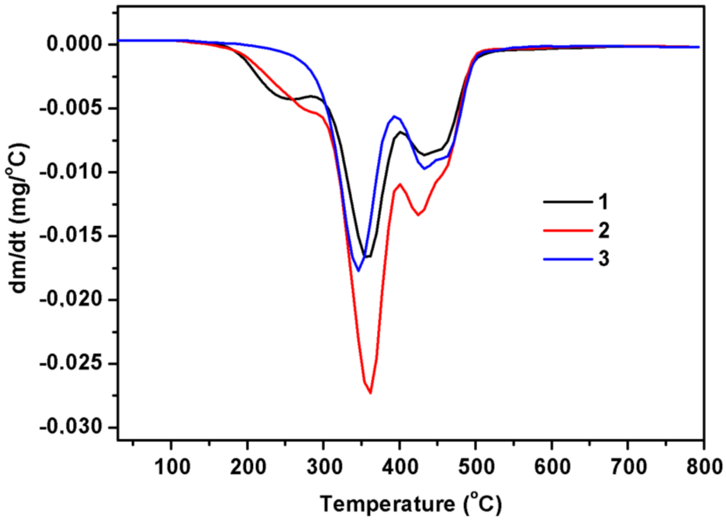

Figure S4. Derivative TG curves of the of the **1**, **2** and **3** cross-linked materials

1.5. UV-vis absorbance of MB and the calibration curves

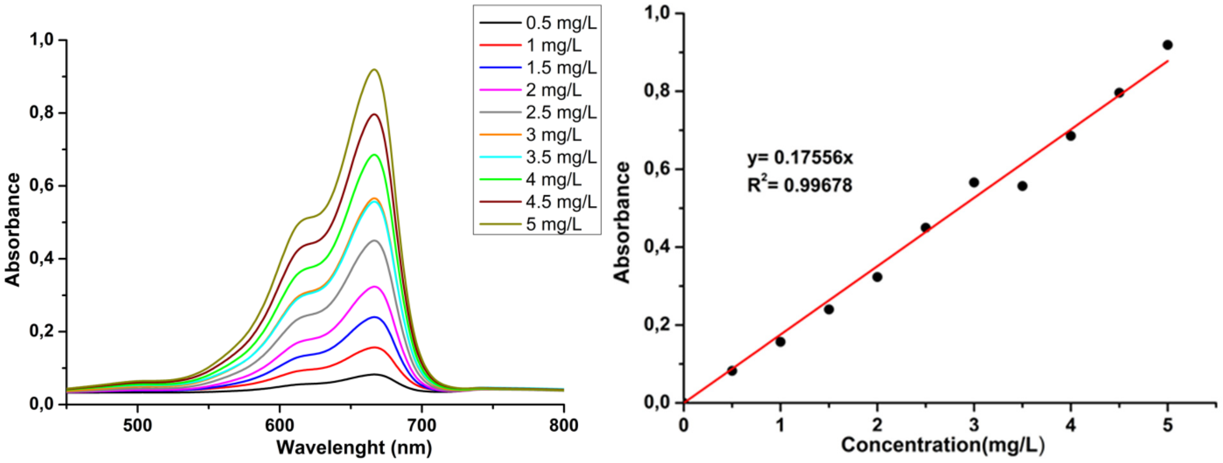

Figure S5. UV-vis absorbance of MB solutions in water (left) and the graphical representation of calibration curve at 667nm (right).

1.6. Desorption process of MB from the sorbent in DMF

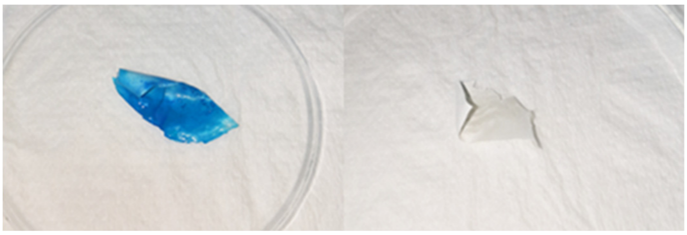

Figure S6. The images of the **3** composite materials after 240 h (left) and after desorption process in DMF (right).

1.7. The 2D AFM topography of the 3 composite after the third cycle of adsorption-desorption

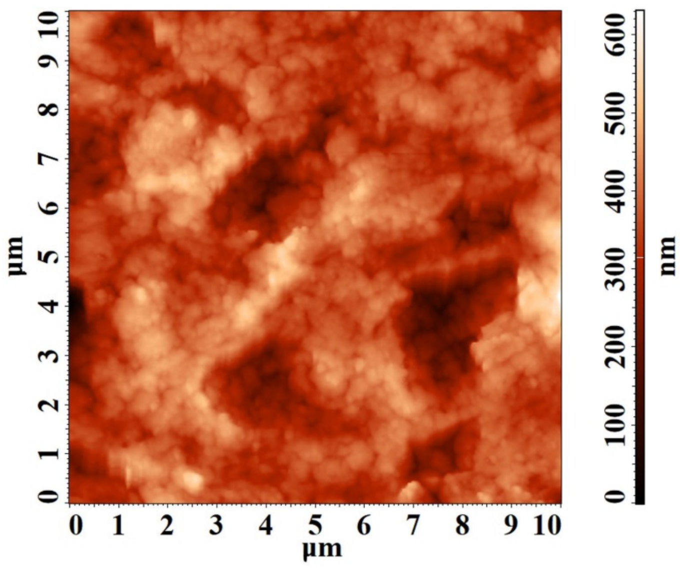

Figure S7. 2D AFM surface topography over of 10 × 10 µm^2 area of **3** composite material after three cycles of adsorption/desorption of MB.

Table S1. AFM surface roughness parameters of **3** composite materials after three cycles of adsorption/desorption of MB.

**Sample**	**S_a (nm)**	**S_q (nm)**	**S_{sk}**	**S_{ku}**	**S_z (nm)**
**3**	54.53	70.82	-0.34	0.65	316.87

S_a = average roughness; S_q = root-mean-square roughness; S_{sk} = skewness of the topography height distribution; S_{ku} = kurtosis of the topography height distributions; S_z = 10-point average of the absolute heights.

1.8. WAXS pattern of the 3 composite after the third cycle of adsorption-desorption

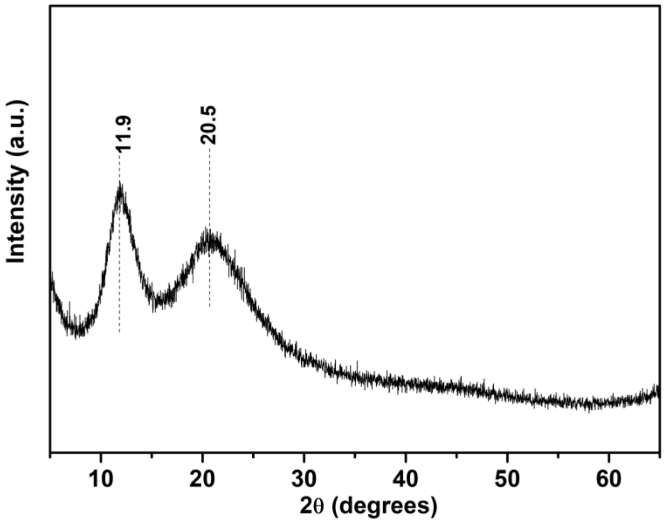

Figure S8. WAXS pattern of **3** composite material after three cycles of adsorption-desorption of MB.
